# Correction to: Liver Disease in Alpha-1 Antitrypsin Deficiency: Current Approaches and Future Directions

**DOI:** 10.1007/s40139-018-0164-z

**Published:** 2018-01-23

**Authors:** Ellen L. Mitchell, Zahida Khan

**Affiliations:** 1 Division of Pediatric Gastroenterology, Hepatology, and Nutrition, Children’s Hospital of Pittsburgh of UPMC, 4401 Penn Avenue, Faculty Pavilion 6th Fl, Pittsburgh, PA 15224-1334 USA; 2 Department of Pediatrics, University of Pittsburgh School of Medicine, Pittsburgh, PA USA; 3 Department of Pathology, University of Pittsburgh School of Medicine, Pittsburgh, PA USA; 4 McGowan Institute for Regenerative Medicine, University of Pittsburgh School of Medicine, Pittsburgh, PA USA; 5 Pittsburgh Liver Research Center, University of Pittsburgh School of Medicine, Pittsburgh, PA USA

**Correction to: Curr Pathobiol Rep (2017) 5(3):243–252**

10.1007/s40139-017-0147-5

The article Liver Disease in Alpha-1 Antitrypsin Deficiency: Current Approaches and Future Directions, written by Ellen L. Mitchell and Zahida Khan, was originally published Online First without open access. After publication in volume 5, issue 3, page 243–252 the author decided to opt for Open Choice and to make the article an open access publication. Therefore, the copyright of the article has been changed to ©The Author(s) 2018 and the article is forthwith distributed under the terms of the Creative Commons Attribution 4.0 International License (http://creativecommons.org/licenses/by/4.0/), which permits use, duplication, adaptation, distribution and reproduction in any medium or format, as long as you give appropriate credit to the original author(s) and the source, provide a link to the Creative Commons license, and indicate if changes were made.

